# Listening for Alzheimer’s clues: machine learning analysis of multidomain speech features for cognitive impairment screening

**DOI:** 10.3389/fnagi.2026.1816747

**Published:** 2026-05-04

**Authors:** Josep Blazquez-Folch, Berta Calm, Adrián Hinojosa-Calleja, Fernando García-Gutiérrez, Montserrat Alegret, Nathalia Muñoz, Amanda Cano, Maria Victoria Fernández, Andrea Miguel, Ariadna Solivar, Itziar De Rojas, Alejandro Valenzuela-Seba, Pablo García-González, Raquel Puerta, Clàudia Olivé, Maria Capdevila-Bayo, Álvaro Muñoz-Morales, Paula Bayón-Buján, Laura Montrreal, Adelina Orellana, Gemma Ortega, Pilar Sanz-Cartagena, Maitee Rosende-Roca, Yahveth Cantero-Fortiz, Miren Jone Gurruchaga, Lluis Tarraga, Christopher Butler, Xavier Montalban, Mercè Boada, Agustín Ruiz, Marta Marquié, Sergi Valero

**Affiliations:** 1 Ace Alzheimer Center Barcelona - Universitat Internacional de Catalunya, Barcelona, Spain; 2 Networking Research Center on Neurodegenerative Diseases (CIBERNED), Instituto de Salud Carlos III, Madrid, Spain; 3 PhD Program in Biotechnology, Faculty of Pharmacy and Food Sciences, University of Barcelona, Barcelona, Spain; 4 Department of Pharmacology, Toxicology and Therapeutic Chemistry, Faculty of Pharmacy and Food Science, University of Barcelona, Barcelona, Spain; 5 Department of Brain Sciences, Imperial College London, London, United Kingdom; 6 The George Institute for Global Health, School of Public Health, Imperial College London, London, United Kingdom; 7 Department of Neurology and Multiple Sclerosis Center of Catalonia (Cemcat), Vall d’Hebron University Hospital, Universitat Autònoma de Barcelona (UAB), Barcelona, Spain; 8 Glenn Biggs Institute for Alzheimer’s and Neurodegenerative Diseases, University of Texas Health Science Center, San Antonio, TX, United States; 9 Department of Microbiology, Immunology and Molecular Genetics, Long School of Medicine, University of Texas Health Science Center, San Antonio, TX, United States

**Keywords:** Alzheimer’s disease, cerebrospinal fluid, early diagnosis, machine learning, mild cognitive impairment, neuropsychological tests, screening

## Abstract

**Introduction:**

Early detection of Alzheimer’s disease (AD) is critical for timely intervention, particularly during the mild cognitive impairment (MCI) stage. This study aimed to develop and evaluate a multidomain speech analysis framework to support cognitive screening, biomarker prediction within the amyloid, tau and neurodegeneration (ATN) framework, and estimation of cognitive function across the AD continuum.

**Methods:**

This study analyzed speech from 2,320 individuals spanning the cognitive spectrum-including those with subjective cognitive decline (SCD), MCI, and Alzheimer’s disease dementia (ADD)-using three spoken tasks (∼3 min) and extracted multidomain features including acoustic, lexical, syntactic, and semantic features. Machine learning models were trained to classify cognitive status, predict amyloid, tau and neurodegeneration (ATN) biomarker positivity, and estimate scores across six neuropsychological domains.

**Results:**

Multidomain speech models achieved high performance in differentiating cognitive stages, with AUC values of up to 0.94 for SCD vs. ADD and 0.82 for SCD vs. MCI classifications. In biomarker prediction, the models yielded AUCs of 0.71, 0.74, and 0.73 for ATN classification, respectively. Speech-based models also showed strong correlations (up to 0.83) with cognitive function scores. Feature importance analysis revealed that verbal fluency measures were the most predictive. Explainability analyses indicated minimal dependency on age, sex, or education, supporting model fairness.

**Discussion:**

These findings show that multidomain speech features capture clinically and biologically relevant information across the AD continuum, enabling cognitive classification, biomarker prediction, and cognitive estimation. These results underscore the potential of speech analysis as a non-invasive, accessible tool for scalable cognitive screening and early detection of AD. These results underscore the potential of speech analysis as a non-invasive, accessible tool for scalable cognitive screening and early detection of AD.

## Introduction

Alzheimer’s disease (AD) and other dementias affect more than 55 million individuals worldwide (World Health Organization [WHO], 2024). Approximately 39.5% of dementia cases in older adults remain undiagnosed (Amjad et al., 2018), while only 8% of older Americans living with mild cognitive impairment (MCI) receive a formal diagnosis (Liu et al., 2024). This lack of early detection is concerning, as individuals with MCI are at a significantly higher risk of developing AD, with annual conversion rates ranging from 3 to 15% (Liu et al., 2023). The consequences of underdiagnosis include delayed access to potentially beneficial treatments, reduced opportunities for care planning, and greater emotional, physical, and financial burdens on individuals and their caregivers (Power et al., 2023; World Health Organization [WHO], 2024). Thus, improving screening, detection, and early diagnosis of cognitive impairment is a critical public health priority to mitigate these risks and improve patient and family outcomes.

AD manifests through progressive cognitive decline, typically impacting memory, language, executive functions, and behavior. These impairments are rooted in the accumulation of amyloid-β (Aβ) plaques and neurofibrillary tangles of phosphorylated tau, detectable through neuroimaging and fluid biomarkers and resulting neurodegeneration (Alzheimer’s Association, 2025; Molinuevo et al., 2018; Peña-Casanova et al., 2012). Neuropsychological batteries have traditionally served as the primary assessment tool for detecting cognitive impairment (Alegret et al., 2012; Espinosa et al., 2013). However, these evaluations often require the physical presence of specialized clinicians in memory clinics, making them resource-intensive and time-consuming. In response to these challenges, numerous online and remote cognitive screening protocols have been proposed to expand accessibility (Alegret et al., 2024; Ding et al., 2022; Meier et al., 2021).

In recent years, digital technologies have revolutionized the landscape of detecting cognitive decline, introducing scalable and effective solutions. Automated speech analysis, in particular, has emerged as a promising tool in detecting AD at its earliest stages (García-Gutiérrez et al., 2023; García-Gutiérrez et al., 2024; Martínez-Nicolás et al., 2021). Language deficits, often evident years before AD progresses to the dementia stage, provide valuable insights into MCI (Sperling et al., 2013; Szatloczki et al., 2015). Speech-based diagnostics leverage advanced machine learning (ML) and natural language processing techniques to analyze linguistic and paralinguistic features from simple speech tasks (Toth et al., 2018). This approach not only facilitates non-invasive and remote longitudinal assessments but also aligns with the growing trend toward e-health technologies and decentralized clinical trials (Tröger et al., 2022).

However, despite the rapid growth of speech-based approaches for cognitive impairment detection, several methodological and translational gaps remain. Prior studies have demonstrated the utility of individual feature domains—including acoustic parameters (Xue et al., 2021), lexical and syntactic measures (Fraser et al., 2016; Pekkala et al., 2013), semantic and graph-based representations (Ambadi et al., 2021), and deep learning–based language embeddings (Amini et al., 2023; Lindsay et al., 2021)—for discriminating cognitive status. Multidomain and multimodal frameworks have also been proposed (Chou et al., 2024; Hajjar et al., 2023; He et al., 2023; Nyongesa et al., 2025), and recent work has explored associations between speech features and amyloid burden in both cognitively unimpaired individuals and those with subjective cognitive decline (Mueller et al., 2021; Verfaillie et al., 2019), as well as longitudinal prediction of AD progression (Amini et al., 2024). Nevertheless, many studies are limited by relatively small or homogeneous cohorts, focus on a restricted set of linguistic domains, lack biomarker-confirmed stratification, or provide limited interpretability of ML models. Furthermore, while amyloid prediction from speech has been explored, comprehensive modeling of the ATN (amyloid, tau, neurodegeneration) framework using multidomain spontaneous speech features remains underdeveloped.

Building on this broader literature, the present study introduces a unified multidomain speech analysis protocol that integrates acoustic, lexical, syntactic, and semantic features within a biomarker-characterized cohort spanning the AD continuum. While previous investigations—including our own—have demonstrated the value of acoustic features for cognitive stage discrimination and amyloid status prediction (García-Gutiérrez et al., 2023; García-Gutiérrez et al., 2024), the current work extends prior approaches in several meaningful ways. First, it systematically incorporates multiple linguistic domains within a single analytical framework to evaluate their complementary diagnostic value. Second, it evaluates prediction not only of clinical status but also of ATN biomarker profiles, thereby aligning speech-based modeling with contemporary biological definitions of AD. Third, it applies advanced feature-selection and optimization strategies to enhance model robustness and interpretability in a larger and more diverse sample. By integrating multidomain linguistic analysis with biomarker-informed stratification and explainable machine-learning techniques, this study aims to advance speech-based digital biomarkers toward clinically scalable, biologically grounded tools for early AD detection.

## Materials and methods

### Study participants

This study included data from individuals evaluated at the Memory Clinic of Ace Alzheimer Center Barcelona between March 2022 and February 2024 (Table 1). Patients were referred to the Memory Clinic by their General Health Practitioner due to subjective memory complaints, or attended the Open House Initiative without a prior physician referral (Abdelnour et al., 2017).

**TABLE 1:** Clinical and sociodemographic characteristics of the sample stratified by diagnosis.

Variable	SCD	naMCI	aMCI	ADD	Non-AD Dementia
Sample size (n, %)	249 (10.7)	465 (20.0)	744 (32.1)	682 (29.4)	180 (7.8)
Age (mean, SD)	66.4 (10.2)	72.4 (10.0)	75.2 (8.5)	80.8 (6.4)	80.7 (7.0)
Sex (% female)	63.1	66.0	60.5	67.9	51.7
Years of formal education (mean, SD)	12.4 (4.1)	9.4 (4.6)	8.4 (4.5)	7.4 (4.5)	6.7 (4.3)
MMSE (mean, SD)	29.3 (0.9)	28.2 (1.7)	26.2 (2.8)	21.7 (3.4)	22.8 (3.5)

SCD, subjective cognitive decline; naMCI, non-amnestic mild cognitive impairment; aMCI, amnestic mild cognitive impairment; ADD, Alzheimer’s disease dementia; AD, Alzheimer’s disease; MMSE, Mini–Mental State Examination; SD, standard deviation.

All participants underwent a series of neurological, neuropsychological, and social evaluations at Ace. The clinical assessment included the Neuropsychological Battery of Fundació Ace (NBACE) (Alegret et al., 2012), the Spanish version of the Mini-Mental State Examination (MMSE) (Blesa et al., 2001; Folstein et al., 1983), the memory test of the Spanish version of the 7 min screening neurocognitive battery (del Ser et al., 2006; Solomon and Pendlebury, 1998), the short form of the Neuropsychiatric Inventory Questionnaire (NPI-Q) (Boada et al., 2006), the Hachinski’s Ischemia Scale (Hachinski et al., 1974), the Blessed Dementia Rating Scale (Blessed et al., 1968), and the Clinical Dementia Rating (CDR) (Morris, 1993). For each participant, the final diagnosis was determined by a multidisciplinary team of neurologists, neuropsychologists, and social workers in a diagnostic consensus conference (Boada et al., 2014).

The cohort included individuals with different degrees of cognitive impairment: (1) SCD (CDR = 0) which refers to the self-perception of cognitive problems, including memory loss, without impairment on standardized cognitive test (Jessen et al., 2014), (2) MCI (CDR = 0.5), which refers to cognitive complaints with impairment on standardized cognitive test according to age and educational level and preserved autonomy (Petersen, 2004), (3) AD dementia (ADD) (CDR > 0.5) (McKhann et al., 2011), and (4) non-AD dementia (CDR > 0.5). Within the MCI group, patients presented an amnestic (aMCI) or a non-amnestic (naMCI) profile (Espinosa et al., 2013). Within the ADD and non-AD dementia groups, patients were divided into mild (CDR = 1), or moderate dementia (CDR = 2). Within the non-AD dementia group, patients presented vascular dementia, Lewy body dementia or frontotemporal dementia behavioral variant. Group differences in continuous variables were assessed using the Kruskal–Wallis test. When significant, pairwise comparisons were performed using Dunn’s post hoc test with Bonferroni correction for multiple testing. Statistical significance was set at *p* < 0.05.

A subset of patients with a diagnosis of MCI underwent lumbar puncture (LP) for the assessment of AD-core biomarkers in CSF. All clinical and biomarker measures were obtained within a 6-month window from the speech protocol administration.

### Neuropsychological data

To assess cognitive performance, the neuropsychological tests from the NBACE battery were organized into several cognitive domains (Alegret et al., 2012). Neuropsychological composites were estimated using structural equation modeling, guided by exploratory factor analysis and expert consensus. Seven composites were analyzed: memory, attention, visuospatial/visuoperception, executive functions, language, orientation, and praxis. This study employed the neuropsychological tests, their domain groupings, and composite estimation as previously described elsewhere (Alegret et al., 2024; García-Gutiérrez et al., 2024).

### Lumbar puncture and quantification of cerebrospinal fluid ATN biomarkers

LPs were conducted at Ace Alzheimer Center Barcelona by an experienced neurologist under fasting conditions. The procedure adhered to the guidelines outlined by the Alzheimer’s Biomarkers Standardization Initiative (Vanderstichele et al., 2012). CSF samples were passively collected using 10 mL polypropylene tubes (Sarstedt Ref 62.610.018) min processed within 2 h of acquisition by centrifugation (2,000 × g for 10 min at 4°C). Following centrifugation, the samples were divided into aliquots and stored in polypropylene tubes (Sarstedt Ref 72.694.007) at −80°C for later analysis. On the analysis day, a 0.5 mL aliquot was thawed to measure Aβ1-42, Aβ1-40, P-tau181, and T-tau levels. These protein concentrations were determined using a chemiluminescence enzyme immunoassay with the Lumipulse G 600 II automated platform (Fujirebio Europe, Göteborg, Sweden) (Leitão et al., 2019). Thresholds established by the CSF program at Ace Alzheimer Center Barcelona defined amyloid positivity as an Aβ1-42/Aβ1-40 ratio below 0.069; and tau (T) positivity as a P-tau181 over 54; and neurodegeneration (N) positivity as a T-tau above 412 pg/mL (Orellana et al., 2022).

### Speech data collection and preprocessing

Audio was recorded using the built-in microphone of the 8th-generation iPad at a standard sampling rate (44.1 kHz) and stored in digital format. Recordings were performed in Spanish in a quiet clinical setting under supervision, minimizing environmental noise and ensuring consistent acquisition conditions across participants. The protocol included three tasks: (1) participants were presented with The Cookie Theft Picture (Cummings, 2019) and they were asked to provide a detailed description of the image (IMG task); (2) a semantic verbal fluency test was conducted where participants were requested to list as many animals as possible within 1-min (SVF task); (3) participants were asked to relate a positive personal experience from the past month (PPE task).

To further characterize the audio quality and recording conditions, several signal-level metrics were computed across all recordings. Summary statistics for recording duration and signal-to-noise ratio, as well as transcription-derived token counts, are reported in Supplementary Table 1.

Subsequently, the audio recordings from these three tasks were resampled to 16 kHz and automatically transcribed using the Whisper large-v2 speech-to-text model (Radford et al., 2022). Subsequently, the stanza (v1.8.2) library was applied on the resulting transcriptions to perform the part-of- speech tagging (POS-tagging) by assigning different lexical categories to each of the transcription tokens (Qi et al., 2020). The generated audio, the transcriptions, and their annotations represented the base information used to extract the features described in the following section (section 2.5).

### Multidomain speech features extraction

Speech features were extracted from the audio data (described in section 2.4) and grouped into five domain categories: acoustic, lexical, syntactical, semantic, and task-specific features. The following sections detail the computation of the features used for the different modeling tasks.

**Acoustic**

Acoustic features enable the characterization of alterations in voice production and paralinguistic aspects such as prosody or speech-related traits. In this study, acoustic features were extracted using the Python libraries OpenSmile (v2.5.0) (Eyben et al., 2010) and librosa (v0.10.2) (McFee et al., 2015).

First, different statistics (mean, coefficient of variation, quartiles, interquartile range, average of positive and negative slopes, skewness, and kurtosis) were calculated from the low-level descriptors (LLDs) of the eGeMAPs feature set (Eyben et al., 2016). A symmetric moving average of three frames was used to compute the LLDs using frames of 20 ms. Among the LLDs used were frequency-related parameters such as the frequency and bandwidth of the first three formants, which are directly related to speech production; the logarithm of the fundamental frequency starting at 27.5 Hz (F0), which represents pitch; and jitter, which measures the variability of the period of the F0. In addition, energy/amplitude parameters such as shimmer, loudness, and harmonics-to-noise ratio, all related to speech quality, were considered. Moreover, spectral parameters such as the relative energy of the first three formants, the alpha ratio, the Hammarberg index, spectral slopes (0–500 Hz and 500–1,500 Hz), harmonic differences (H1-H2 and H1-A3), spectral flux, and the first four Mel-frequency cepstral coefficients were calculated.

Second, using the librosa library, mean, standard deviation, skewness, and kurtosis statistics were computed for several acoustic parameters, using librosa default parameters. The analyzed features included chromagram characteristics, root mean square, zero crossing rate of the signal, tempo, onset envelope, spectral bandwidth, and spectral centroid.

All of the described features were calculated for voiced segments, except for loudness, which was computed across the entire audio. Alpha ratio, spectral slopes (0–500 Hz and 500–1,500 Hz), and Hammarberg index were calculated for both voiced and unvoiced segments. Additionally, temporal parameters such as the mean, standard deviation, and quartiles of the voiced and unvoiced segments were included in the acoustic parameter set. The open-source speaker-diarization-3.1 model from pyannote (Bredin et al., 2020) was employed to perform voice activity detection.

**Lexical**

Lexical features characterize the use of linguistic elements related to vocabulary and word usage, two attributes affected by cognitive impairment (Ahmed et al., 2013; Fraser et al., 2016; Garrard et al., 2005). Collectively, these features quantify the richness of information communicated in speech. In this study, lexical features were calculated using POS-tagging information and word frequency norms (Speer, 2022).

Based on POS-tagging information, the ratios of different lexical categories were considered by calculating the proportions of nouns, verbs, adjectives, adverbs, auxiliaries, and adpositions relative to the total number of words. Additionally, ratios between specific lexical categories, such as adjectives to nouns, adjectives to verbs, and nouns to verbs, were considered (Ahmed et al., 2013). Other metrics, such as the measure of textual lexical diversity (MTLD) as presented in (McCarthy and Jarvis, 2010), the moving-average type-token ratio (MATTR), and the Uber, Guiraud, MassLog, and Herdan indices, were calculated (Covington and McFall, 2010; Kurdi, 2023). The MTLD was computed using thresholds of 0.65, 0.70, and 0.75, and it was recalculated considering only nouns, verbs, adjectives, and adverbs. The MATTR was calculated using a window size of 10 tokens with a five-token stride, considering only nouns, verbs, adjectives, and adverbs.

Based on word frequency norms, using the Python library wordfreq (v3.1.1) (Speer, 2022), the mean and standard deviation of the logarithm of word frequency excluding functional words were determined.

Features based on POS-tagging information were calculated only for the IMG and PPE tasks, while those based on word frequency were applied to all tasks.

**Syntactical**

The syntactic features evaluate the structure and complexity of spoken sentences, providing information on the grammatical organization of the discourse. In this study, scores were extracted from the dependency and constituency trees using stanza (v1.8.2) (Qi et al., 2020) and based on the universal dependencies format (Nivre et al., 2020).

From the constituency-based trees, the Yngve and Frazier indices were calculated, as well as the maximum and average depths of the generated syntactic trees (Voleti et al., 2020). For each metric and task, the average value of the indices for all sentences was considered. Alternatively, for the dependency-based trees, the maximum and average number of root dependencies across all sentences were computed (Voleti et al., 2020). Syntactic features were computed only for the IMG and PPE tasks.

**Semantic**

This group of features includes metrics that analyze the conceptual information of the words used in speech. Within the group of semantic features, norm-based and data-driven features were considered.

First, based on published Spanish norms of word age of acquisition (Alonso et al., 2015), the mean and standard deviation of word age of acquisition in each patient’s speech were computed. These features were extracted for all tasks, providing a baseline measure of linguistic maturity.

Second, for the IMG and SVF tasks, word embeddings were computed using a word2vec model (Mikolov et al., 2013) trained ad hoc with transcription data from our database, excluding functional words. Subsequently, various features were extracted from the generated word embeddings to analyze the richness and complexity of the semantic elements of the discourse. Specifically, the Euclidean distance and cosine similarity of the word embeddings were considered, as well as the mean and standard deviation of the pairwise distances between words. Additionally, spectral clustering was performed on the entire vocabulary, stratified by task, and each word was assigned a cluster membership. This analysis allowed us to examine the number of clusters visited by each subject, the number of transitions between clusters, the number of words within each cluster, the frequency of repetition of previously visited clusters, and the Euclidean distance and cosine similarity between pairs of visited clusters. Overall, these metrics provide insight into the semantic coherence and diversity of the language.

The Python library gensim (v4.3.0) (Rehurek and Sojka, 2011) was used for training the word2vec models. For spectral clustering, Scikit-Learn (v1.5.1) (Pedregosa et al., 2012) was employed.

**Task-specific features**

In addition, specific features were extracted by exploiting the unique characteristics of each speech task.

For the IMG task, the features proposed in Ambadi et al. (2021), based on spatial semantic graphs, were used. These features aim to characterize the participant’s visual narrative during the image description. Specifically, the following metrics were calculated: the number of content information units (CIUs) mentioned, the distance between different CIUs, the mean and standard deviation of the distance traveled on the vertical and horizontal axes, the mean number of words used to describe each CIU, the proportion of keywords related to the CIUs relative to the total number of words (concreteness), the number of times a participant repeated the same CIU (both consecutively and non-consecutively), the number of times the participant moved between two CIUs within the same quadrant of the image, the proportion of times the participant moved between CIUs in different quadrants versus the same quadrant, and the ratio of CIUs mentioned in each quadrant to the total number of CIUs in the quadrant.

Relative to the SVF task, the following features were considered: the total number of animals mentioned, the number and proportion of unique animals to the total, the proportion of non-animals, the number of repeated animals, and the sum and mean number of animals between repeated animals.

Finally, given the properties of the PPE task, sentiment features were extracted using the model presented in Pérez et al. (2023). The sentiments considered were a positive, negative, or neutral description, and the detection of emotions such as sadness, happiness, anger, disgust, fear, and surprise.

### Machine learning modeling

**FIGURE 1:**
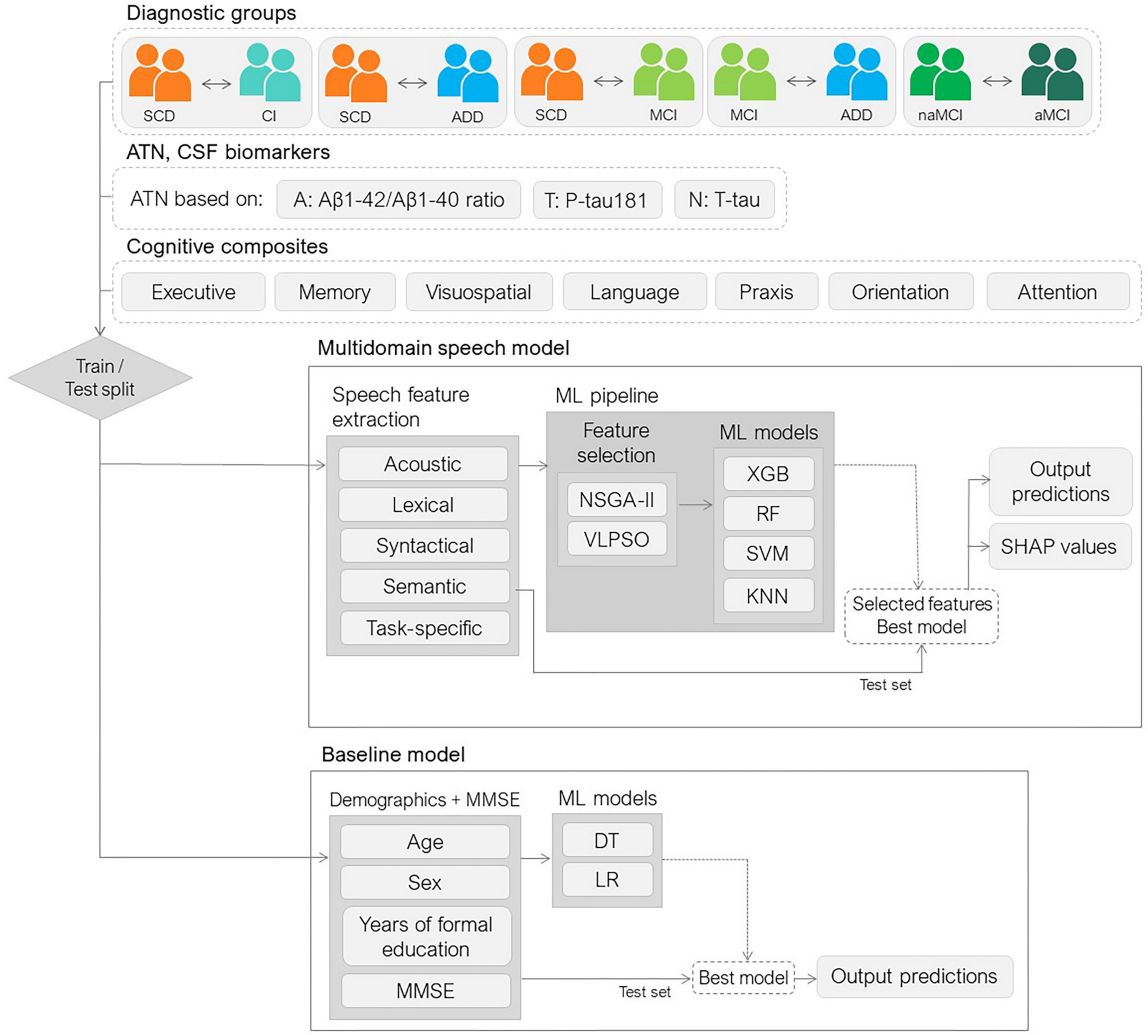
Multidomain speech and baseline model framework. The dataset was divided into training and testing sets to evaluate two approaches: a multidomain speech model and a baseline clinical model. The speech model incorporated features from linguistic and paralinguistic domains, while the baseline model relied on demographic information and cognitive screening scores. SCD, subjective cognitive decline; CI, cognitive impairment; ADD, Alzheimer’s disease dementia; MCI, mild cognitive impairment; naMCI, non-amnestic mild cognitive impairment; aMCI, amnestic mild cognitive impairment; ATN, amyloid, tau, and neurodegeneration; CSF, cerebrospinal fluid; ML, machine learning; NSGA-II, non-dominated sorting genetic algorithm II; VLPSO, variable-length particle swarm optimization; XGB, eXtreme Gradient Boosting; RF, random forest; SVM, support vector machine; KNN, k-nearest neighbors; SHAP, SHapley Additive exPlanations; MMSE, Mini–Mental State Examination; SD, standard deviation; DT, decision tree; LR, logistic regression.

Using the feature set described in section 2.5, ML models were trained for both classification and regression tasks. The framework used is summarized in Figure 1.

Classification tasks focused on diagnosing different cognitive conditions included: cognitive impairment (CI) detection (SCD vs. MCI and dementia), ADD identification (SCD vs. ADD; and MCI vs. ADD), MCI detection (SCD vs. MCI), and MCI subtype discrimination (naMCI vs. aMCI). Also, classification models were used for the prediction of ATN status.

Regression tasks aimed to predict cognitive composite values (as detailed in section 2.2).

The data were divided into training (80%) and testing sets (20%), stratified by diagnostic condition, with subjects matched for age, sex, and education within each set. Differences between training and test sets within each diagnostic group were assessed using Mann–Whitney U tests for continuous variables and chi-square tests for sex. Bonferroni correction was applied to account for multiple comparisons (*p* < 0.05). No significant differences were observed between training and test sets across diagnostic groups for demographic and clinical variables (all Bonferroni-corrected *p* > 0.05), supporting the adequacy of the data split. All model development steps (feature selection and hyperparameter optimization) were performed exclusively on the training set, while the test set was held out and used only for the final evaluation of the models. Furthermore, to evaluate the predictive ability of each speech test, models were trained for each task using each test independently (IMG, SVF, or PPE) and combined (IMG + SVF + PPE).

The models employed for each problem included Random Forest (RF), eXtreme Gradient Boosting (XGB), and Support Vector Machine (SVM). For all tasks and models, an initial feature selection step was conducted based on the training data. The well-established non-dominated sorting genetic algorithm II (NSGA-II) (Deb et al., 2002) was used for feature selection, optimizing the balanced accuracy (BA) for classification tasks and the mean absolute error (MAE) for regression tasks, along with the number of selected features (Garcia-Gutierrez et al., 2022; Xue et al., 2016). Additionally, the variable-length particle swarm optimization (VLPSO) (Tran et al., 2019) was used for feature selection in the ATN status prediction as it showed the best performance in this task in previous works (García-Gutiérrez et al., 2023). The performance metrics to be optimized were evaluated on the validation set using five-fold cross-validation (CV). An SVM was used as classification/regression model within the NSGA-II and k-nearest neighbors (KNN) were used within the VLPSO.

After feature selection, the hyperparameters of all models were optimized using Bayesian optimization with a tree-structured Parzen estimator as the surrogate model (Bergstra et al., 2011). During this optimization, the BA and MAE were maximized or minimized respectively, using the training set, and optimizing the metrics using the validation data from a 5-fold CV. A total of 1,000 iterations were performed in the hyperparameter optimization, with the first 500 iterations conducted randomly.

After completing feature selection and hyperparameter optimization, the final models were retrained on the full training set and evaluated on the independent test set. Performance on the test set reflects the generalization ability of the final model on unseen data.

Additionally, ML models trained on multidomain speech features were compared to baseline screening models trained on MMSE and Demo (demographics: age, sex, and years of formal education). The ML models used for baseline models were the decision tree and logistic regression.

All models were developed in Python (v3.11.10). The Scikit-Learn (v1.2.2) (Pedregosa et al., 2012) implementation was used for the RF and SVM models. For the XGB algorithm, XGBoost (v2.1.1) (Chen and Guestrin, 2016) was adopted. The pymoo (v0.6.1.3) (Blank and Deb, 2020) library was used to implement the NSGA-II, the pywinEA2 (available on GitHub) was used to implement the VLPSO and hyperparameter optimization was carried out using optuna (v3.6.1) (Akiba et al., 2019). For more details on the hyperparameters and the search space of each model, refer to Supplementary Appendix 2.

### Machine learning explainability

For each task, the best-performing model was selected. Subsequently, explainable AI (XAI) techniques were employed to assess the interpretability of the models.

SHapley Additive exPlanations (SHAP) values were used to gain insights into model predictions and feature importance. SHAP values were computed for the test set to explain individual predictions by attributing importance to each feature highlighting the most influential features (Lundberg and Lee, 2017). To aid interpretation at a higher level, SHAP feature importance values were grouped by feature domain. However, since larger groups can accumulate more importance by virtue of having more features, we normalized the total SHAP importance of each group by dividing by the number of features it contained. This adjustment ensures comparability between groups of different sizes and reduces the bias favoring larger groups (Gregorutti et al., 2015).

Finally, a sex-based analysis examined whether models were prone to sex-based errors. A chi-square test compared sex in the original dataset and misclassified samples. Biases related to age and year of formal education were assessed using a t-test. To explore sex-based feature importance differences, SHAP values were grouped by feature domain, aggregated per sex, and compared using t-tests to assess statistical significance between men and women.

## Results

### Study participants

This study included 2,320 patients evaluated at the Memory Clinic of Ace Alzheimer Center Barcelona: 249 patients with SCD (CDR = 0), 1,209 patients with MCI (CDR = 0.5) (Petersen, 2004), and 862 patients with dementia (646 with mild dementia, CDR = 1.0; and 216 with moderate dementia, CDR = 2.0). Within the MCI group, 744 were aMCI, while the remaining 465 were naMCI. Among the dementia group, 682 were diagnosed with probable/possible ADD, 117 with vascular dementia, 41 with Lewy body dementia, and 22 with Frontotemporal dementia behavioral variant. A clear age and MMSE gradient were observed within groups, with SCD patients being the youngest (66.4 ± 10.2 years; MMSE 29.3 ± 0.9) and ADD patients the oldest (80.8 ± 6.4 years; MMSE 21.7 ± 3.4), concomitant with a decline in years of formal education (from 12.4 ± 4.1 in SCD to 7.4 ± 4.5 in ADD dementia). The clinical and sociodemographic characteristics of participants are summarized in Table 1.

Significant differences across diagnostic groups were observed for both age and MMSE (Kruskal–Wallis test, *p* < 0.001 for both variables). *Post hoc* pairwise comparisons with Bonferroni correction revealed significant differences between all groups for MMSE, while age differences were significant between all groups except between ADD and non-AD dementia (Supplementary Appendix 3).

A subsample of 236 patients with MCI underwent LP for the assessment of AD-core biomarkers in CSF. Their demographic and clinical characteristics are summarized in Table 2 stratified by ATN status. 52.5% of cases were A+, 55.9% were T+, and 46.2% were N+.

**TABLE 2:** Mean comparison of clinical and sociodemographic features stratified by ATN status.

Variable	A-	A+	T-	T+	N-	N+
Sample size (n, %)	112 (47.5)	124 (52.5)	104 (44.1)	132 (55.9)	127 (53.8)	109 (46.2)
*APOE* ε4 carriers (%)	13.8	50.0	18.3	43.6	22.1	45.0
Age (mean, SD)	69.7 (9.0)	76.2 (5.1)	69.4 (8.9)	76.0 (5.4)	70.9 (8.7)	75.7 (5.9)
Sex (% female)	51.7	63.7	51.9	62.8	50.3	66.9
Years of formal education (mean, SD)	8.7 (4.2)	8.3 (4.8)	9.0 (4.5)	8.1 (4.5)	8.8 (4.6)	8.2 (4.4)
MMSE (mean, SD)	27.3 (2.6)	25.6 (2.8)	27.2 (2.7)	25.8 (2.8)	27.1 (2.7)	25.7 (2.8)

A, Aβ1-42/Aβ1-40 ratio; T, P-tau181; N, T-tau; MMSE, Mini–Mental State Examination; SD, standard deviation.

### Discrimination between the diagnostic groups

**FIGURE 2:**
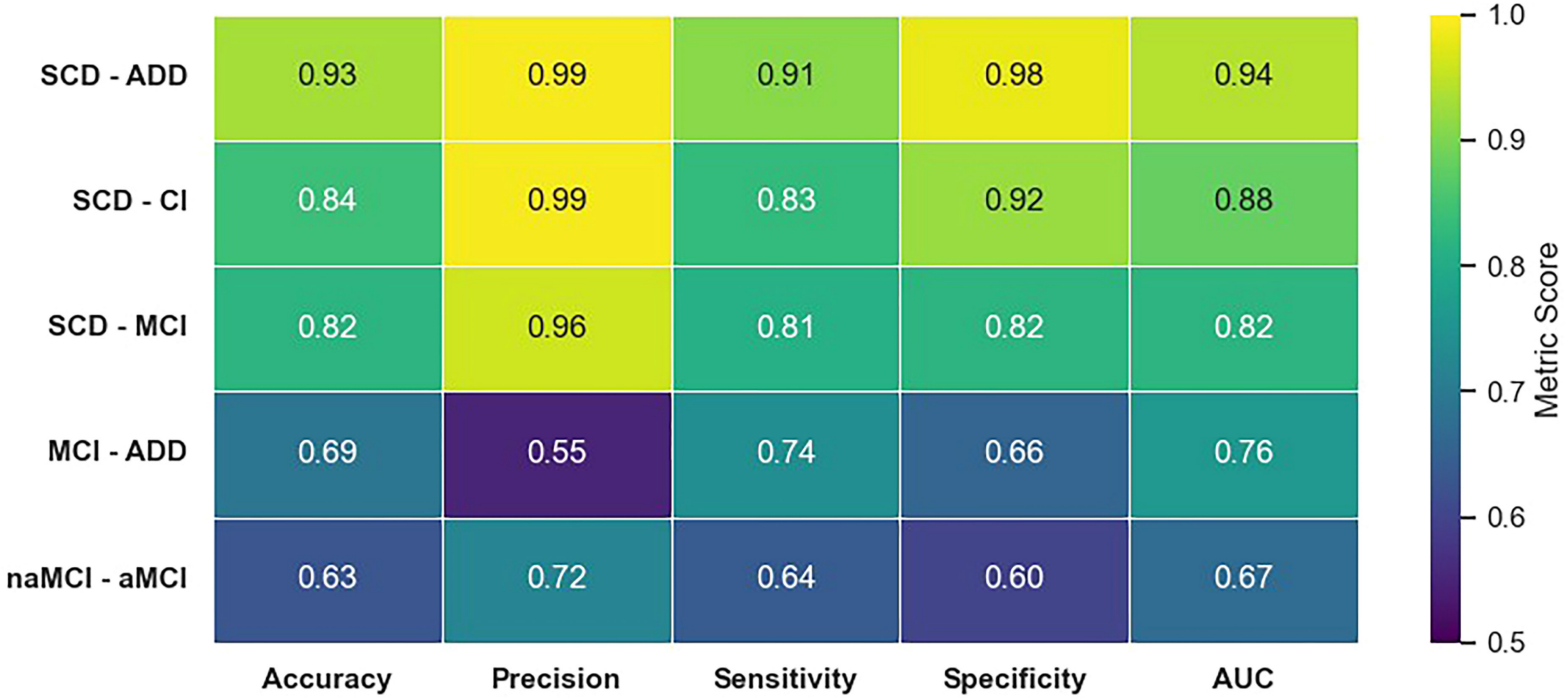
Diagnosis prediction performance metrics using the best performing models trained on multidomain speech feature set. SCD, subjective cognitive decline; ADD, Alzheimer’s disease dementia; CI, cognitive impairment; MCI, mild cognitive impairment; naMCI, non-amnestic mild cognitive impairment; aMCI, amnestic mild cognitive impairment; AUC, area under the curve.

Among the multidomain speech models, the highest sensitivity and specificity were achieved in discriminating SCD-ADD, followed by SCD-CI and SCD-MCI. In contrast, the naMCI-aMCI and MCI-ADD comparisons presented the greatest challenges for diagnostic classification using speech data (Figure 2).

For the SCD-ADD and MCI-ADD discriminations, the baseline model trained on the MMSE+Demo feature set achieved a higher AUC than the multidomain speech models. In contrast, for the SCD-CI, SCD-MCI, and naMCI-aMCI classifications, the multidomain speech models yielded superior AUC. For SCD-ADD discrimination, the baseline model achieved nearly perfect prediction, with an accuracy of 0.99 with an AUC of 0.99. Similarly, speech-based models demonstrated robust performance, with a 0.94 AUC (Supplementary Appendix 4).

When relying solely on multidomain speech features, combining features from all three tasks consistently outperformed the use of individual task features. Additionally, when evaluating individual tasks, models utilizing SVF-generated features consistently achieved higher BA than those based on IMG and PPE features, with PPE performing worse than both SVF and IMG (Supplementary Appendix 4).

### Cognitive domains prediction

**FIGURE 3:**
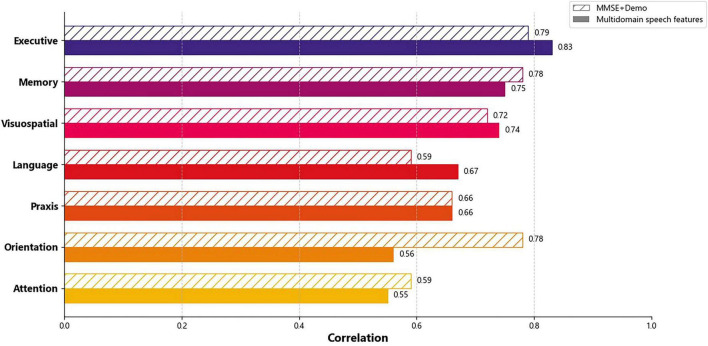
Cognitive domains prediction correlation of baseline models (MMSE+Demo) against the best performing multidomain speech features models. MMSE, Mini–Mental State Examination; Demo, demographics.

The baseline model trained on the MMSE+Demo feature set outperformed the multidomain speech models in predicting memory, attention, and orientation cognitive composites. Conversely, the multidomain speech models excelled in predicting the executive, visuospatial, and language cognitive composites. Correlation between the predicted values and cognitive composites further revealed that the best results were achieved for the executive cognitive composite, followed by memory, visuospatial, and language composites (Figure 3).

Models that were trained using a combination of multidomain speech features from all three tasks exhibited higher correlations and greater coefficient of determination (R2) than models trained on individual tasks. Within the individual tasks, SVF-generated features generally outperformed those derived from IMG and PPE, except in predicting the visuospatial neuropsychological cognitive composite, where IMG task features yielded better results. PPE consistently produced the worst performance among the three tasks (Supplementary Appendix 4).

### ATN detection

The detection of CSF biomarker positivity yielded AUC values ranging from 0.71 to 0.74 when using the multidomain speech feature set. The MMSE+Demo feature set only outperformed the speech models in predicting A+ cases (Table 3).

**TABLE 3:** Discrimination of ATN status.

ATN status	Feature set	Model	Acc	Pre	Sen	Spe	AUC
**A+/A-**	MMSE+Demo	LR	0.77	0.81	0.81	0.7	0.76
	Multidomain speech (SVF)	VLPSO+XGB	0.69	0.72	0.81	0.5	0.71
**T+/T-**	MMSE+Demo	DT	0.79	0.79	0.91	0.56	0.73
	Multidomain speech (IMG)	VLPSO+KNN	0.77	0.75	0.97	0.39	0.74
**N+/N-**	MMSE+Demo	DT	0.65	0.63	0.86	0.42	0.64
	Multidomain speech (IMG)	VLPSO+KNN	0.69	0.8	0.57	0.83	0.73

A, Aβ1-42/Aβ1-40 ratio; T, P-tau181; N, T-tau; Acc, accuracy; Pre, precision; Sen, sensitivity; Spe, specificity; AUC, area under the curve; MMSE, Mini–Mental State Examination; Demo, demographics; LR, logistic regression; DT, decision tree; VLPSO, variable-length particle swarm optimization; XGB, eXtreme gradient boosting; KNN, K-nearest neighbors; SVF, semantic verbal fluency test; IMG, image description task.

The most effective models employed VLPSO feature selection rather than NSGA-II. In contrast to the findings on previous sections, for the prediction of ATN status, models using multidomain speech features from a single task achieved higher BAs compared to those combining features across tasks. Specifically, the model trained on SVF features was the best performing for the A+/A- discrimination, while the IMG feature set yielded the best results for TN detection (Table 3).

### Machine learning explainability results

**FIGURE 4:**
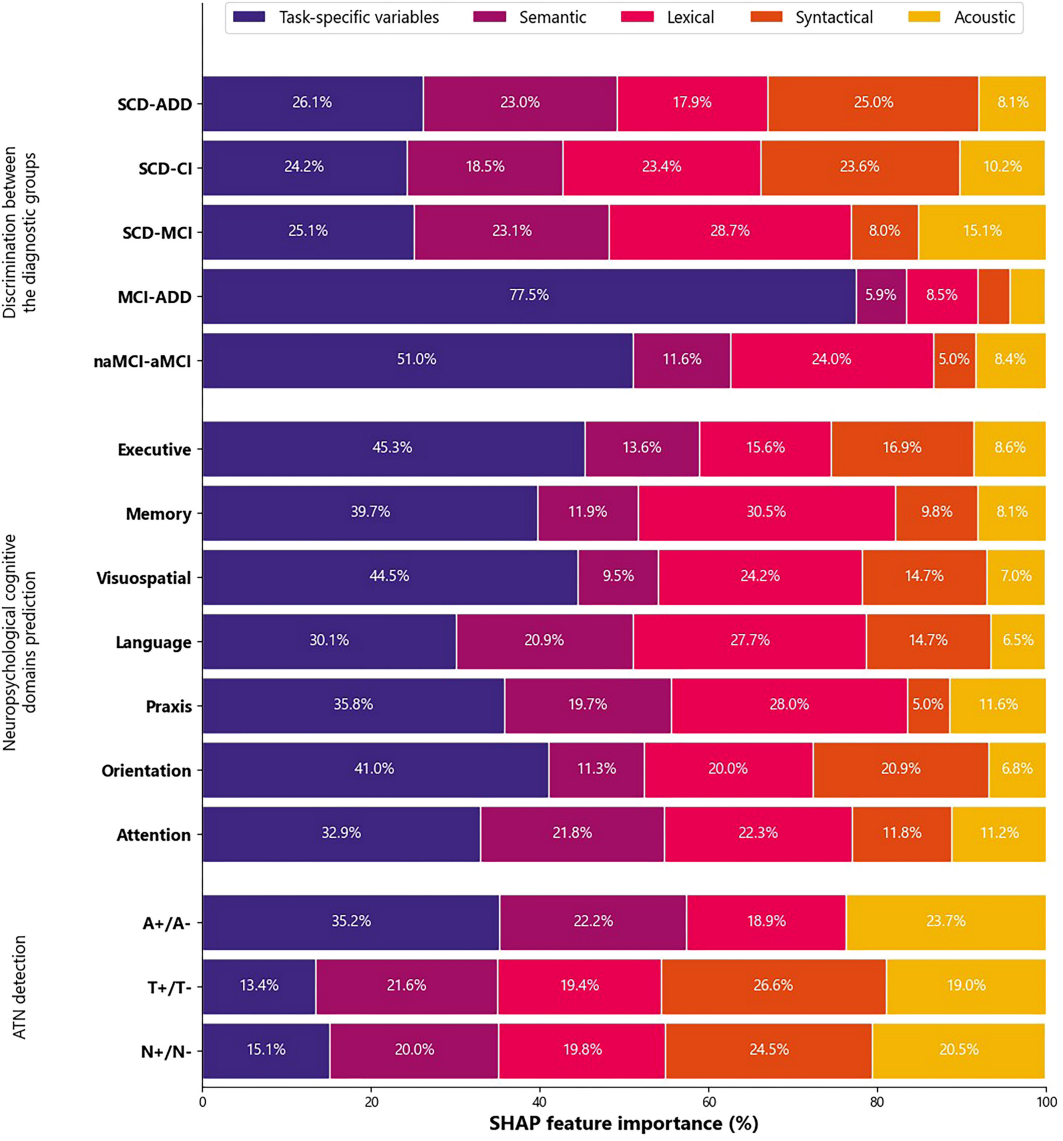
SHAP feature importance by feature domain for the different tasks. SCD, subjective cognitive decline; ADD, Alzheimer’s disease dementia; CI, cognitive impairment; MCI, mild cognitive impairment; naMCI, non-amnestic mild cognitive impairment; aMCI, amnestic mild cognitive impairment; A, Aβ1-42/Aβ1-40 ratio; T, P-tau181; N, T-tau.

Features from multiple domains generally exhibited balanced importance across most diagnostic classification tasks, with the notable exception of MCI-ADD discrimination. In this case, task-specific features—particularly the number of unique animals named in the SVF task—accounted for the majority of predictive importance. For cognitive domain prediction, task-specific features remained the most salient features within the speech domain. Notably, the number of unique animals in the SVF task emerged as the most influential feature across all neuropsychological composite predictions. In contrast, for ATN status classification, acoustic features played a more prominent role in determining the predicted label. Nevertheless, linguistic features still contributed the largest share of feature importance (Figure 4). Notably, for ATN detection, no individual feature stands out.

Most models were not prone to bias related to sex, age, or years of formal education. A sex-based analysis revealed no significant tendency toward sex-related misclassification, with the exception of the MCI-ADD model, which was prone to sex-based error, showing a higher proportion of women in misclassified samples (75.4%) compared to the original dataset (64.9%). Age-related bias was observed in three models: SCD- CI and SCD-MCI were prone to age-related errors with misclassified samples having a lower mean age than the original dataset, while T-tau model showed the opposite trend. Only the SCD-CI model was prone to bias based on years of formal education.

When comparing SHAP features aggregated by domain, none of the *t*-tests showed a statistically significant difference between sexes (all *p* > 0.05).

## Discussion

This study investigated the potential of multidomain speech features for the detection of cognitive impairment and AD. The findings demonstrate that speech-based models, leveraging acoustic, lexical, syntactic, and semantic features, can effectively differentiate between cognitive stages and predict the presence of CSF biomarkers associated with AD pathology.

The applied ML models showed a good discriminatory capacity of speech parameters for distinguishing among the diagnostic groups (Figure 2) and predicting ATN status in CSF (Table 3). Also, ML models were able to estimate cognitive performance using speech features (Figure 3). Furthermore, by incorporating XAI techniques, we gained valuable insights into how different features influenced the decisions made by the models (Figure 4).

Our study confirms prior evidence suggesting that speech impairment manifests early in AD progression and can be systematically captured through automated speech analysis. In our previous works (García-Gutiérrez et al., 2023; García-Gutiérrez et al., 2024), we relied solely on a restricted set of paralinguistic features. Previous research has shown that linguistic deficits, particularly in lexical retrieval and fluency, correlate with early cognitive decline, reinforcing the utility of linguistic biomarkers in cognitive impairment screening (Gomez and White, 2006; Pekkala et al., 2013). As demonstrated by other researchers, using a more diverse feature set derived from speech analysis could substantially enhance the performance of predictive models (Amini et al., 2023; Mahajan and Baths, 2021; Xue et al., 2021).

The multidomain speech model used in our work achieved competitive performance in distinguishing diagnosis stages of cognitive impairment. On the identification of the ADD diagnosis (SCD-ADD and MCI-ADD), the baseline model using MMSE and demographics outperformed the speech model. Nevertheless, on all the other discrimination pairs, the speech model was superior to the proposed baseline model. The classification metrics obtained were consistent with previous studies (Amini et al., 2023; Asgari et al., 2017; Chen et al., 2021; Gosztolya et al., 2021; He et al., 2023; Lindsay et al., 2021; Mahajan and Baths, 2021; Themistocleous et al., 2018; Xue et al., 2021). Therefore, our findings provide new evidence supporting the potential of automated speech analysis for the identification of different stages of AD. Also, it was shown that the combination of speech features from multiple speech tasks improves prediction performance.

Cognitive tests from a standardized battery of neuropsychological measures were grouped into seven composite scores representing the cognitive domains of attention, executive functions, language, memory, visuospatial functions, orientation, and praxis (Alegret et al., 2012). Using these cognitive composites as the target variables of the ML models, the correlations obtained with speech parameters were higher than in previous works in all cognitive domains (García-Gutiérrez et al., 2024). Notably, executive function and memory demonstrated particularly strong associations with speech parameters. In contrast, the attention domain (though affected in AD) showed weaker correlations with speech biomarkers, consistent with prior findings (García-Gutiérrez et al., 2024).

During the preclinical stage of AD, before symptom onset, the pathophysiological course of the disease is characterized by first the formation of A plaques and later p-tau protein aggregates, which accumulate in the brain and disrupt normal neuronal function (Sperling et al., 2013). Subsequently, at the MCI stage, the accumulation of these proteins in the brain reaches a critical threshold, leading to neuronal injury and pathological changes in the volumes of different brain regions (Sperling et al., 2013). The main idea here is that the analysis of speech and language could provide relevant information about the underlying pathophysiological process of AD (Voleti et al., 2020). Our results align with studies using AD-related biomarkers conducted by Hajjar et al. (2023) in a longitudinal cohort of cognitively unimpaired individuals and MCI patients, García-Gutiérrez et al. (2023) in a cross-sectional study of MCI subjects, Mueller et al. (2021) in a longitudinal study involving healthy individuals and early-stage MCI patients, and Verfaillie et al. (2019) in a cross-sectional study of individuals with cognitive decline. These studies demonstrated that amyloid burden is associated with several speech parameters. Notably, the studies of Mueller et al. (2021) and Verfaillie et al. (2019) were based on the lexico-syntactic content of the speech, while García-Gutiérrez et al. (2023) focuses on the properties of the sound generated when describing a picture. We extended the previous studies to the detection of ATN status providing a new landmark for the use of speech biomarkers in neurodegenerative disorders (Table 3).

Feature importance analysis in our study illustrates that all the speech domains played a dominant role in diagnostic classification. The number of unique animals listed in the SVF task emerged as the strongest predictor across some tasks, underscoring the role of semantic richness. Importantly, our explainability analyses revealed that the models exhibited minimal dependency on sociodemographic variables such as age, sex, or years of formal education. With the exception of a few tasks, no statistically significant biases were observed, and the SHAP-based feature domain importance did not differ between sexes. This suggests that our speech-based models possess strong potential for generalizability across diverse populations. The low reliance on demographic variables also supports the notion of democratization in cognitive assessment—highlighting that such models could be deployed in broader, more heterogeneous settings without exacerbating existing health disparities.

A key advantage of our approach is its integration of multiple linguistic and acoustic dimensions, surpassing prior studies focused on isolated speech domains. While some existing research has explored the diagnostic potential of single-domain speech features (Ambadi et al., 2021; García-Gutiérrez et al., 2024; Kurdi, 2023), our findings suggest that a multidimensional approach enhances predictive accuracy and robustness. This comprehensive framework allows for a more holistic assessment of cognitive function and neurodegeneration. A key strength of this approach is the systematic assessment of model biases, a critical requirement for ensuring the reliability and fairness of ML applications in healthcare.

Despite these promising results, several limitations must be acknowledged. Although our cohort is relatively large and diverse, external validation using independent datasets is necessary to confirm the generalizability of our findings. Additionally, the study is based on cross-sectional observations, which may not fully capture the dynamic and multifaceted relationship between dementia and multidomain speech features. Longitudinal studies are essential to understand how speech patterns evolve over time in parallel with amyloid and tau pathology (Amini et al., 2024). Another limitation is the exclusive focus on Spanish-speaking participants; therefore, future research should explore the applicability of these models across different languages and cultural contexts (Lindsay et al., 2021). This would help ensure that speech-based diagnostic tools are robust, generalizable, and inclusive. While our models achieved good discrimination for many tasks, there is still room for improved precision in distinguishing MCI subtypes and predicting biomarker positivity. Further refinement of feature selection methods and model architectures could enhance predictive performance (Gosztolya et al., 2021).

Building on the current framework, future studies could benefit from incorporating neuroimaging data into the modeling process. Early alterations in brain metabolism and functional connectivity—particularly in language-related areas, have been linked to subtle speech changes in preclinical AD, suggesting that multimodal approaches may offer improved diagnostic precision (Scheltens et al., 2021). Moving beyond a cross-sectional design is essential, and longitudinal analyses of speech-based biomarkers could provide a clearer view of how language features evolve over time, support tracking of disease progression across the AD spectrum, and help distinguish AD pathology from other types of dementia (Amini et al., 2024). Another crucial aspect is the potential of remote and passive speech-based screening and monitoring for neurodegenerative diseases (Popp et al., 2024). Given the feasibility of deploying these models in telemedicine settings, speech biomarkers could bridge accessibility gaps in early AD detection.

Given the advancements in AI and ML, speech biomarkers could become a key tool in early intervention strategies for AD. Future work should explore the integration of AI-driven speech analysis with other digital health solutions to develop a standardized framework for cognitive screening and disease monitoring. The potential to deploy such methods in telehealth applications could revolutionize early detection and patient management strategies, paving the way for more personalized and timely interventions in neurodegenerative disorders.

## Conclusion

In conclusion, this study highlights the significant potential of multidomain speech analysis as a non-invasive and accessible method for detecting and monitoring AD and cognitive impairment. By integrating acoustic, lexical, syntactic, and semantic features, our approach offers a robust predictive framework. The strong correlation between speech-derived biomarkers and neurophysiological functions underscores the viability of speech analysis as a scalable screening tool.

Furthermore, the effectiveness of this approach in detecting cognitive changes across the AD continuum, as well as its ability to identify early signs of amyloidosis and tauopathy, supports its potential for early diagnosis and intervention. While our findings are promising, further research is needed to validate these results in diverse population cohorts and to refine predictive models for greater accuracy. Future studies should also explore the integration of speech-based assessments into telemedicine platforms to enhance accessibility and incorporate AI-driven solutions for automated cognitive screening.

The convergence of ML, natural language processing, and digital health presents a transformative opportunity to improve early detection and monitoring of AD. By addressing current challenges and optimizing speech-based methodologies, this research contributes to the development of innovative and widely applicable diagnostic solutions for neurodegenerative diseases, ultimately paving the way for more personalized interventions and improved patient outcomes.

## Data Availability

The datasets generated and analyzed during the current study are not publicly available as they contain human privacy-sensitive data. The code used in this study will also not be shared. However, the datasets are available from the corresponding author upon reasonable request.
