# Supplementary material for: Listening for Alzheimer’s clues: machine learning analysis of multidomain speech features for cognitive impairment screening
Source: Front Aging Neurosci. 2026 May 4;18:1816747. doi: 10.3389/fnagi.2026.1816747 (PMC13180916; doi:10.3389/fnagi.2026.1816747)
Supplement: Supplementary file 1 [file Data_Sheet_1.docx]

**Appendix**

**Appendix A.** **Descriptive Statistics of Audio Recordings**

This appendix presents detailed descriptive statistics of the audio recordings across diagnostic groups (Table A1).

**Appendix B. Machine learning models and feature selection algorithms hyperparameters**

This appendix details the hyperparameters of all the ML models used in the main manuscript. Table B2 lists the hyperparameters of the algorithms used for the classification and regression problems. Table B3 summarizes the hyperparameters of the feature selection algorithms.

**Appendix C.**

**Fig C1.** Distribution of age and MMSE across diagnostic groups. Boxplots illustrate the distribution of age (left) and MMSE scores (right) for each diagnostic group (SCD, naMCI, aMCI, ADD, and non-AD dementia). Boxes represent the interquartile range (IQR), horizontal lines indicate the median, whiskers extend to 1.5× IQR, and points denote outliers. Significant differences between groups were observed for both variables (Kruskal–Wallis test, p < 0.001), with Bonferroni-corrected post hoc comparisons confirming differences between all groups for MMSE and between all groups for age except between ADD and non-AD dementia.

**Appendix D. Machine learning model results**

This appendix summarizes the results obtained by the classification (Table D4) and regression models (Table D5).

**Table A1.** Descriptive statistics of audio recordings across diagnostic groups. Values are presented as mean and SD for total recording duration (seconds), SNR (dB), and number of words derived from Whisper-based automatic transcriptions, reported for the full recording and for each individual task. Total duration reflects the concatenation of the three tasks (IMG, SVF, PPE).

|  |  | SCD | naMCI | aMCI | ADD | Non-AD Dementia |
| --- | --- | --- | --- | --- | --- | --- |
| Length (mean, SD) | Whole audio | 140.24 (26.73) | 139.24 (26.20) | 142.50 (27.11) | 145.12 (26.00) | 146.24 (23.72) |
|  | IMG | 41.86 (14.71) | 43.96 (15.23) | 44.30 (15.46) | 46.79 (15.37) | 47.71 (14.67) |
|  | SVF | 63.64 (2.09) | 63.63 (2.20) | 63.89 (2.01) | 63.82 (2.14) | 63.51 (2.13) |
|  | PPE | 34.75 (16.98) | 31.65 (16.36) | 34.31 (17.45) | 34.52 (17.21) | 35.02 (15.94) |
| SNR (mean, SD) | Whole audio | 24.14 (3.43) | 24.73 (4.07) | 24.53 (4.01) | 25.13 (4.35) | 25.34 (4.40) |
|  | IMG | 23.72 (3.45) | 24.57 (4.10) | 24.53 (3.97) | 25.26 (4.56) | 25.28 (4.57) |
|  | SVF | 23.34 (4.11) | 23.48 (4.50) | 23.25 (4.58) | 23.84 (4.84) | 24.43 (4.90) |
|  | PPE | 21.03 (3.49) | 22.56 (3.94) | 22.83 (4.14) | 23.61 (4.40) | 24.07 (4.48) |
| Number of words (mean, SD) | Whole audio | 185.13 (61.41) | 162.45 (59.50) | 167.67 (64.73) | 174.13 (64.37) | 158.19 (60.98) |
|  | IMG | 77.85 (30.97) | 68.54 (29.74) | 67.01 (29.71) | 67.81 (29.07) | 59.06 (25.16) |
|  | SVF | 31.99 (11.46) | 30.65 (15.17) | 34.91 (19.18) | 43.44 (24.25) | 36.86 (20.72) |
|  | PPE | 75.29 (39.31) | 63.26 (36.36) | 65.75 (39.94) | 62.87 (38.62) | 62.27 (38.00) |

Abbreviations: SCD: subjective cognitive decline; naMCI: non-amnestic mild cognitive impairment; aMCI: amnestic mild cognitive impairment; ADD: Alzheimer’s disease dementia; AD: Alzheimer’s disease; SNR: Signal-to-Noise ratio; SD: standard deviation; IMG: image description task; SVF: semantic verbal fluency task; PPE: positive personal experience task.

**Table B2.** Hyperparameters of classification and regression models.

| Model | Parameter ^a^ | Value/search space ^b^ |
| --- | --- | --- |
| Support vector machines | Class weight | Balanced |
|  | Kernel | Radial basis function |
|  | C | [10^-5^, 10^2^] |
|  | γ | [10^-5^, 10^2^] |
| Random forest | Number of estimators | 200 |
|  | Class weight ^c^ | Balanced subsample |
|  | Max depth | {2,7} |
|  | Min samples split | [0.01, 0.2] |
|  | Min samples leaf | [0.01, 0.2] |
|  | Max features | [0.5, 1.0] |
|  | Max samples | [0.5, 1.0] |
|  | CCP α | [10^-6, 0.5] |
|  | Criterion ^d^ | Squared error |
| XGBoost | Number of estimators | 200 |
|  | Max depth | {2,12} |
|  | Learning rate | [10^-2^, 0.3] |
|  | γ | [10^-6^, 100] |
|  | Min child weight | [0, 100] |
|  | Subsample | [0.2, 1.0] |
|  | colsample_bytree | [0.2, 1.0] |
|  | colsample_bynode | [0.2, 1.0] |
|  | L1 regularization | [0.1, 10] |
|  | L1 regularization | [0.1, 10] |
| Decision tree | Class weight ^c^ | Balanced |
|  | Max depth | {2,7} |
|  | Min samples split | [0.01, 0.2] |
|  | Min samples leaf | [0.01, 0.2] |
|  | Max features | [0.5, 1.0] |
|  | CCP α | [10^-6^, 0.5] |
|  | Criterion ^d^ | Absolute error |
| K-nearest neighbours | Number of neighbours | {2, 15} |
|  | Weights | {Uniform, Distance} |
| Logistic regression | C | [10^-5^, 1] |

^a^ Hyperparameters not listed in this table were selected at their default value. ^b^ {·} indicates sampling of categorical values, while [·] indicates sampling of real values. ^c^ Parameter only considered for classification problems. ^d^ Parameter only considered for regression problems.

**Table B3.** Hyperparameters of the algorithms used to perform feature selection.

| Method | Parameter ^a^ | Value |
| --- | --- | --- |
| NSGA-II | Generations | 500 |
|  | Population size | 150 |
|  | Sampling | Binary random sampling |
|  | Crossover | Two-point crossover |
|  | Mutation | Bitflip mutation |
| VLPSO | Population size | 150 |
|  | Iterations | 500 |
|  | η | 0.2 |
|  | ω | 0.2 |
|  | γ | 0.5 |
|  | λ | 0.5 |
|  | C_1_ | 0.1 |
|  | C_2_ | 0.2 |
|  | NbrDiv | 10 |
|  | α | 40 |
|  | β | 100 |

^a^ Parameters not listed in this table were selected at their default value. Abbreviations: NSGA-II, Non-dominated Sorting Genetic Algorithm; VLPSO, Variable-Length Particle Swarm Optimization.

**
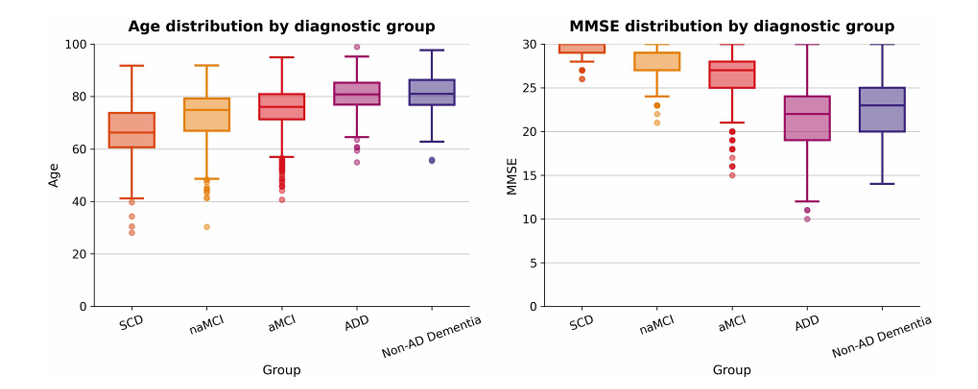
**

**Fig C1.** Distribution of age and MMSE across diagnostic groups. Boxplots illustrate the distribution of age (left) and MMSE scores (right) for each diagnostic group (SCD, naMCI, aMCI, ADD, and non-AD dementia). Boxes represent the interquartile range (IQR), horizontal lines indicate the median, whiskers extend to 1.5× IQR, and points denote outliers. Significant differences between groups were observed for both variables (Kruskal–Wallis test, p < 0.001), with Bonferroni-corrected post hoc comparisons confirming differences between all groups for MMSE and between all groups for age except between ADD and non-AD dementia.

**Table D4.** Discrimination of diagnosis groups.

| Group | Task | Feature set | Model | Acc | Pre | Sen | Spe | AUC |
| --- | --- | --- | --- | --- | --- | --- | --- | --- |
| SCD-CI | - | MMSE+Demo | DT | 0.81 | 0.99 | 0.8 | 0.9 | 0.85 |
|  | IMG | Multidomain speech | NSGA-II+SVM | 0.76 | 0.96 | 0.77 | 0.76 | 0.76 |
|  | SVF | Multidomain speech | NSGA-II+XGB | 0.83 | 0.98 | 0.84 | 0.86 | 0.92 |
|  | PPE | Multidomain speech | NSGA-II+RF | 0.71 | 0.97 | 0.68 | 0.82 | 0.8 |
|  | 3 tasks | Multidomain speech | NSGA-II+SVM | 0.84 | 0.99 | 0.83 | 0.92 | 0.88 |
| SCD-ADD | - | MMSE+Demo | LR | 0.99 | 1 | 0.99 | 1 | 0.99 |
|  | IMG | Multidomain speech | NSGA-II+SVM | 0.81 | 0.96 | 0.78 | 0.9 | 0.84 |
|  | SVF | Multidomain speech | NSGA-II+SVM | 0.93 | 0.98 | 0.93 | 0.94 | 0.93 |
|  | PPE | Multidomain speech | NSGA-II+XGB | 0.82 | 0.92 | 0.83 | 0.8 | 0.87 |
|  | 3 tasks | Multidomain speech | NSGA-II+SVM | 0.93 | 0.99 | 0.91 | 0.98 | 0.94 |
| SCD-MCI | - | MMSE+Demo | DT | 0.77 | 0.92 | 0.8 | 0.64 | 0.72 |
|  | IMG | Multidomain speech | NSGA-II+SVM | 0.71 | 0.93 | 0.69 | 0.76 | 0.73 |
|  | SVF | Multidomain speech | NSGA-II+XGB | 0.77 | 0.96 | 0.75 | 0.86 | 0.87 |
|  | PPE | Multidomain speech | NSGA-II+XGB | 0.79 | 0.91 | 0.83 | 0.58 | 0.77 |
|  | 3 tasks | Multidomain speech | NSGA-II+SVM | 0.82 | 0.96 | 0.81 | 0.82 | 0.82 |
| MCI-ADD | - | MMSE+Demo | LR | 0.84 | 0.8 | 0.75 | 0.9 | 0.82 |
|  | IMG | Multidomain speech | NSGA-II+RF | 0.68 | 0.55 | 0.58 | 0.73 | 0.68 |
|  | SVF | Multidomain speech | NSGA-II+RF | 0.7 | 0.56 | 0.72 | 0.69 | 0.75 |
|  | PPE | Multidomain speech | NSGA-II+SVM | 0.66 | 0.52 | 0.63 | 0.67 | 0.65 |
|  | 3 tasks | Multidomain speech | NSGA-II+RF | 0.69 | 0.55 | 0.74 | 0.66 | 0.76 |
| naMCI-aMCI | - | MMSE+Demo | DT | 0.63 | 0.74 | 0.6 | 0.67 | 0.64 |
|  | IMG | Multidomain speech | NSGA-II+XGB | 0.57 | 0.66 | 0.64 | 0.46 | 0.57 |
|  | SVF | Multidomain speech | NSGA-II+RF | 0.64 | 0.73 | 0.65 | 0.62 | 0.67 |
|  | PPE | Multidomain speech | NSGA-II+SVM | 0.58 | 0.66 | 0.68 | 0.43 | 0.55 |
|  | 3 tasks | Multidomain speech | NSGA-II+RF | 0.63 | 0.72 | 0.64 | 0.6 | 0.67 |

Abbreviations: SCD: subjective cognitive decline; ADD: Alzheimer's disease dementia; CI: cognitive impairment; MCI: mild cognitive impairment; naMCI: non-amnestic mild cognitive impairment; aMCI: amnestic mild cognitive impairment; Acc: accuracy; Pre: precision; Sen: sensitivity; Spe: specificity; AUC: area under the curve; MMSE: Mini–Mental State Examination; Demo: demographics; DT: decision tree; LR: logistic regression; SVM: support vector machine; XGB: eXtreme gradient boosting; RF: random forest; NSGA-II: non-dominated sorting genetic algorithm II; IMG: image description task; SVF: semantic verbal fluency task; PPE: positive personal experience task.

**Table D5.** Neuropsychological cognitive domains prediction.

| Composite | Task | Feature set | Model | MAE | R^2^ | Correlation |
| --- | --- | --- | --- | --- | --- | --- |
| Memory | - | MMSE+Demo | DTR | 0.46 | 0.61 | 0.78 |
|  | IMG | Multidomain speech | NSGA-II+SVM | 0.58 | 0.38 | 0.62 |
|  | SVF | Multidomain speech | NSGA-II+RF | 0.49 | 0.52 | 0.73 |
|  | PPE | Multidomain speech | NSGA-II+XGB | 0.64 | 0.28 | 0.53 |
|  | 3 tasks | Multidomain speech | NSGA-II+XGB | 0.49 | 0.56 | 0.75 |
| Executive | - | MMSE+Demo | LR | 0.43 | 0.62 | 0.79 |
|  | IMG | Multidomain speech | NSGA-II+XGB | 0.5 | 0.47 | 0.68 |
|  | SVF | Multidomain speech | NSGA-II+XGB | 0.42 | 0.64 | 0.8 |
|  | PPE | Multidomain speech | NSGA-II+XGB | 0.58 | 0.34 | 0.59 |
|  | 3 tasks | Multidomain speech | NSGA-II+XGB | 0.38 | 0.69 | 0.83 |
| Attention | - | MMSE+Demo | LR | 0.5 | 0.35 | 0.59 |
|  | IMG | Multidomain speech | NSGA-II+RF | 0.55 | 0.21 | 0.46 |
|  | SVF | Multidomain speech | NSGA-II+RF | 0.53 | 0.26 | 0.51 |
|  | PPE | Multidomain speech | NSGA-II+RF | 0.58 | 0.14 | 0.38 |
|  | 3 tasks | Multidomain speech | NSGA-II+XGB | 0.52 | 0.29 | 0.55 |
| Visuospatial | - | MMSE+Demo | LR | 0.42 | 0.52 | 0.72 |
|  | IMG | Multidomain speech | NSGA-II+SVM | 0.45 | 0.43 | 0.66 |
|  | SVF | Multidomain speech | NSGA-II+XGB | 0.46 | 0.43 | 0.65 |
|  | PPE | Multidomain speech | NSGA-II+XGB | 0.56 | 0.2 | 0.45 |
|  | 3 tasks | Multidomain speech | NSGA-II+XGB | 0.41 | 0.54 | 0.74 |
| Language | - | MMSE+Demo | DTR | 0.41 | 0.31 | 0.59 |
|  | IMG | Multidomain speech | NSGA-II+SVM | 0.44 | 0.3 | 0.56 |
|  | SVF | Multidomain speech | NSGA-II+RF | 0.42 | 0.38 | 0.62 |
|  | PPE | Multidomain speech | NSGA-II+SVM | 0.5 | 0.11 | 0.4 |
|  | 3 tasks | Multidomain speech | NSGA-II+XGB | 0.4 | 0.45 | 0.67 |
| Orientation | - | MMSE+Demo | DTR | 0.24 | 0.6 | 0.78 |
|  | IMG | Multidomain speech | NSGA-II+SVM | 0.41 | 0.16 | 0.44 |
|  | SVF | Multidomain speech | NSGA-II+SVM | 0.38 | 0.25 | 0.53 |
|  | PPE | Multidomain speech | NSGA-II+SVM | 0.42 | 0.11 | 0.39 |
|  | 3 tasks | Multidomain speech | NSGA-II+XGB | 0.38 | 0.31 | 0.56 |
| Praxis | - | MMSE+Demo | DTR | 0.51 | 0.4 | 0.66 |
|  | IMG | Multidomain speech | NSGA-II+SVM | 0.58 | 0.31 | 0.57 |
|  | SVF | Multidomain speech | NSGA-II+SVM | 0.54 | 0.37 | 0.62 |
|  | PPE | Multidomain speech | NSGA-II+XGB | 0.65 | 0.17 | 0.43 |
|  | 3 tasks | Multidomain speech | NSGA-II+SVM | 0.52 | 0.41 | 0.66 |

Abbreviations: MAE: mean absolute error; $R^2$: coefficient of determination; MMSE: Mini–Mental State Examination; Demo: demographics; DTR: decision tree regressor; LR: logistic regression; SVM: support vector machine; XGB: eXtreme gradient boosting; RF: random forest; NSGA-II: non-dominated sorting genetic algorithm II; IMG: image description task; SVF: semantic verbal fluency task; PPE: positive personal experience task.
